# Acidosis promotes invasiveness of breast cancer cells through ROS-AKT-NF-κB pathway

**DOI:** 10.18632/oncotarget.2514

**Published:** 2014-09-25

**Authors:** Subash C. Gupta, Ramesh Singh, Radhika Pochampally, Kounosuke Watabe, Yin-Yuan Mo

**Affiliations:** ^1^ Cancer Institute, University of Mississippi Medical Center, Jackson, MS; ^2^ Department of Biochemistry, University of Mississippi Medical Center, Jackson, MS; ^3^ Department of Microbiology, University of Mississippi Medical Center, Jackson, MS; ^4^ Department of Pharmacology and Toxicology, University of Mississippi Medical Center, Jackson, MS

**Keywords:** Acidosis, AKT, NF-κB, Invasion, breast cancer, inflammation, ROS, tumor microenvironment

## Abstract

It is well known that acidic microenvironment promotes tumorigenesis, however, the underlying mechanism remains largely unknown. In the present study, we show that acidosis promotes invasiveness of breast cancer cells through a series of signaling events. First, our study indicates that NF-κB is a key factor for acidosis-induced cell invasion. Acidosis activates NF-κB without affecting STAT3 activity; knockdown of NF-κB p65 abrogates the acidosis-induced invasion activity. Next, we show that the activation of NF-κB is mediated through phosphorylation and degradation of IκBα; and phosphorylation and nuclear translocation of p65. Upstream to NF-κB signaling, AKT is activated under acidic conditions. Moreover, acidosis induces generation of reactive oxygen species (ROS) which can be suppressed by ROS scavengers, reversing the acidosis-induced activation of AKT and NF-κB, and invasiveness. As a negative regulator of AKT, PTEN is oxidized and inactivated by the acidosis-induced ROS. Finally, inhibition of NADPH oxidase (NOX) suppresses acidosis-induced ROS production, suggesting involvement of NOX in acidosis-induced signaling cascade. Of considerable interest, acidosis-induced ROS production and activation of AKT and NF-κB can be only detected in cancer cells, but not in non-malignant cells. Together, these results demonstrate a cancer specific acidosis-induced signaling cascade in breast cancer cells, leading to cell invasion.

## INTRODUCTION

Majority of cancer deaths are caused by metastatic spread of cells from the primary tumor to a distant site. In order to metastasize, tumor cells first locally invade through surrounding extracellular matrix and stromal cell layers [1]. Tumor invasion and metastasis involve multiple interactions and complex crosstalk between tumor cells and their microenvironment [2]. It has been shown that the extracellular pH (pHe) within the microenvironment of breast tumors is significantly low (acidic) compared with that of normal tissue [3]. The low pHe (acidosis) in tumor microenvironment is mainly caused by a combination of poor vascular perfusion, regional hypoxia, and increased aerobic glycolysis (Warburg effect) and lactic acid production [4, 5]. A previous study has shown that an increase in glucose uptake is associated with the transition from carcinoma *in situ* to invasive breast cancer [6]. In particular, highest regions of tumor invasion correspond to areas with the lowest pHe and tumor invasion does not occur in regions with normal or near normal pHe levels in a nude mouse model [7]. Moreover, oral sodium bicarbonate has been shown to reduce the formation of spontaneous and experimental breast cancer metastases to the lung [8]. These reports suggest that acidosis promotes breast cancer invasion; however, the underlying mechanism still remains elusive.

A key factor responsible for cell invasion is the pro-inflammatory transcription factor, nuclear factor (NF)-κB [9]. NF-κB is a ubiquitously expressed pleiotropic transcription factor that can be activated in response to a number of stimuli including low pHe [10-12]. Under normal conditions, NF-κB stays in the cytoplasm as a heterotrimeric complex consisting of the subunits p50, p65, and the inhibitory subunit IκBα. In response to inducing stimuli, IκB undergoes phosphorylation, ubiquitination and proteolytic degradation and the p65-p50 dimeric complex is then released in the cytoplasm. Next, the p65 subunit undergoes phosphorylation and moves into the nucleus where it binds to specific DNA sequence and activates the transcription of hundreds of genes [13]. The phosphorylation of IκBα is catalyzed by IκBα kinase (IKK), which consists of three subunits, IKK-α, IKK-β, and IKK-γ (also called NEMO). Aberrant regulation of NF-κB and the signaling pathways that control its activity is linked with inflammation, drug/radiation resistance, and tumorigenic potential of cancer cells [14]. However, it is largely unclear how acidosis induces the NF-κB signaling, leading to cell invasion.

In the present work, we report that the activation of NF-κB is essential to acidosis-induced invasiveness of breast cancer cells. Moreover, acidosis induces production of reactive oxygen species (ROS), and activates PDK1 and AKT, leading to NF-κB activation. Finally, we show that this acidosis-mediated ROS-AKT-NF-κB signaling cascade is specific to cancer cells.

## RESULTS

The purpose of this study was to dissect acidosis-mediated signaling pathways, leading to cell invasion in breast cancer. Although most experiments were performed in MDA-MB-231 and MCF-7, other cell lines were also used. Because the extracellular pH within the microenvironment of solid tumors including breast tumors is typically in the range of 6.5-6.9 [15, 16], we adjusted pH of the culture medium to 6.6 with 20 mM 2-(N-morpholino)ethane-sulfonic acid and 20 mM Tris (hydroxymethyl) aminomethane [11].

### Acidosis increases the invasion activity and induces NF-κB activation

**Figure 1:**
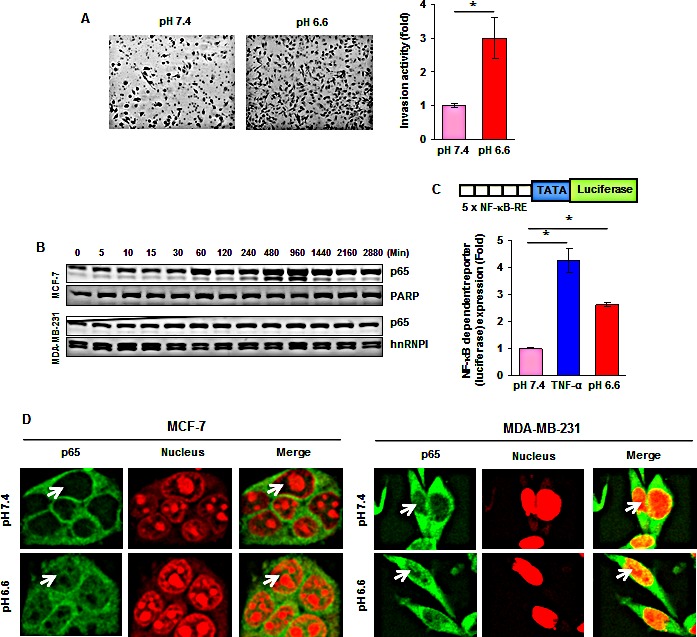
Acidosis increases invasive capacity and NF-κB activity (*A*, *left*) MDA-MB-231 cells were cultured at pH 7.4 or pH 6.6 for 48 hours and then assessed *in vitro* in regular medium using Matrigel invasion chambers. (*A*, *right*) Fold induction in the number of invaded cells at pH 6.6 as compared to those cultured at pH 7.4. (*B*) Time-dependent effects of acidic environment on NF-κB p65 nuclear translocation. Cells were cultured at pH 6.6 for the indicated time, nuclear extracts were prepared and assayed for p65 content by Western blotting. (*C*) Acidic environment induces NF-κB-dependent reporter gene expression in MCF-7 cells. Cells were transiently transfected with a plasmid in which a luciferase reporter carries 5 copies of NF-κB response elements (5 × NF-κB-RE) in front of minimal promoter (TATA). Cells were then cultured at pH 7.4 or pH 6.6 for 12 hours and luciferase activity was measured using whole cell lysate. Where indicated, the cells cultured in normal medium were exposed to 0.5 nM TNF-α for 12 hours. (*D*) Cellular localization of NF-κB p65 in breast cancer cells under acidic environment. Cells were cultured at pH 7.4 or pH 6.6 for 1 hour and analyzed for p65 localization by immunefluorescent staining. *, *p*< 0.05.

First, we investigated if acidosis can affect the invasion activity of breast cancer cells. MDA-MB-231 cells were cultured at pH 7.4 or pH 6.6 for 48 hours and then assessed *in vitro* in regular medium using Matrigel invasion chambers. The invasion activity under acidic conditions was a 3-fold higher than that cultured at pH 7.4 (Fig. 1*A*).

We then measured the activities of the pro-inflammatory transcription factor NF-κB under acidic conditions because NF-κB is a key factor responsible for cell invasion. *First*, we found that low pHe was able to increase the nuclear NF-κB p65 in a time-dependent manner in both MCF-7 and MDA-MB-231 cells (Fig. 1*B*). In contrast, acidosis had no effect on the activities of signal transducer and activator of transcription 3 (STAT3), another pro-inflammatory transcription factor (Fig. S1*A*). *Second*, luciferase reporter assays revealed a 2.6-fold induction of NF-κB activity under acidic conditions (Fig. 1*C*). *Third*, acidosis induced the redistribution of p65 from the cytoplasm to the nucleus, as determined by immunofluorescence staining (Fig. 1*D*). In addition, we found that acidosis induced the NF-κB downstream target CXCR4 which has been implicated in invasion and metastasis (Fig. S1*B*). Since regular culture medium often becomes acidic after cells are grown for a period of time, we replaced with fresh medium after every 10-12 hours. Cells cultured in pH7.4 medium for a period of 1 to 48 hours showed no change of the nuclear NF-κB-p65 (Fig. S1*C*). Collectively, these findings suggest that low pHe can induce NF-κB activation in breast cancer cells.

### Acidosis induces IκBα phosphorylation and degradation

**Figure 2:**
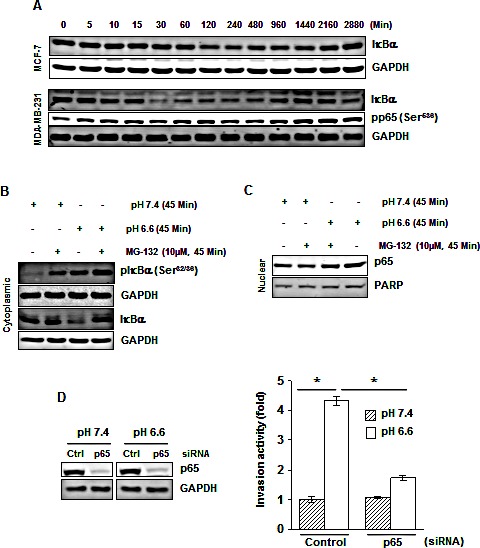
Acidosis induces IκBα phosphorylation, IκBα degradation and p65 phosphorylation (*A*) Cells were cultured at pH 6.6 for the indicated times, cytoplasmic extracts were prepared and analyzed for IκBα and pp65 by Western blotting. (*B*) Acidosis induces IκBα phosphorylation in MDA-MB-231 cells. Cells were cultured at pH 7.4 or pH 6.6 in absence or presence of MG-132 for 45 minutes. Cytoplasmic extracts were analyzed by Western blotting using a phospho-specific IκBα (Ser^32/36^) and IκBα antibodies, and (*C*) Nuclear extracts were analyzed for p65. (*D*) Gene silencing of NF-κB-p65 abolishes acidosis induced invasion activity of MDA-MB-231 cells. (*Left*), cells were transfected with p65 siRNA and control siRNA in normal medium. After 24 h, cells were cultured under normal or acidic medium for 24 hours, and whole-cell extracts were analyzed by Western blotting using p65 antibody. (*Right*), the p65 silenced cells cultured under normal and acidic conditions were assessed *in vitro* for invasion activity using Matrigel chambers. *, *p*< 0.05.

Under normal conditions, IκB, the inhibitory subunit of NF-κB, holds NF-κB in an inactive state in the cytoplasm. The translocation of NF-κB to the nucleus is preceded by the phosphorylation, ubiquitination, and proteolytic degradation of IκBα. We examined whether acidosis-induced NF-κB activation is mediated through IκBα phosphorylation and degradation. Cells were cultured in low pH medium for a period of 5 to 2880 minutes (48 hours) and cytoplasmic fraction was used to examine IκBα expression. We found that acidosis induced IκBα degradation in both MCF-7 and MDA-MB-231 cells (Fig. 2*A*), although the kinetics of degradation was different in two cell lines; IκBα was resynthesized after 16 hours (Fig. 2*A*). We then investigated whether acidosis induces IκBα phosphorylation. Cells were exposed to normal or low pH medium in the presence of a proteasome inhibitor (MG-132) and Western blot was carried out using an antibody that recognizes the phosphorylated form of IκBα (Ser^32^ and Ser^36^). Results revealed that acidosis did induce phosphorylation of IκBα (Fig. 2*B*). The densitometric analyses using National Institutes of Health Image J software indicated that the acidosis-induced nuclear content of p65 was reduced by 19% in the presence of proteasome inhibitor (Fig. 2*C*). Overall, these results suggest that acidosis-induced NF-κB activation is mediated through phosphorylation and degradation of IκBα.

### Knockdown of NF-κB p65 abrogates acidosis-induced invasion activity

To determine whether NF-κB activation plays a crucial role in acidosis-induced invasion activity, we silenced p65 by RNAi (Fig. 2*D*, *left*) before culturing MDA-MB-231 cells under acidic conditions followed by invasion assay. Acidosis-induced invasion activity of MDA-MB-231 cells was decreased from a 4.3-fold to a 1.7-fold when cells were transfected with p65 siRNA (Fig. 2*D*, *right*). These results suggest that an increase in invasion activity under acidic conditions is mediated through NF-κB activation.

### Acidosis induces AKT activation

**Figure 3:**
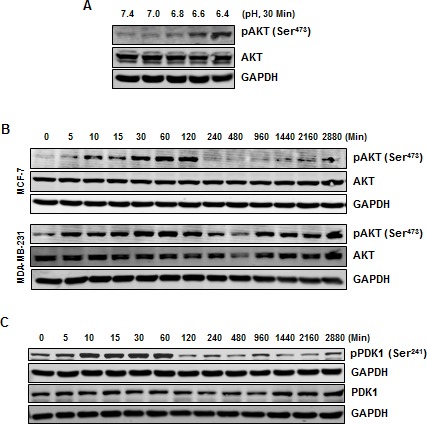
Acidosis induces phosphorylation of AKT and PDK1 (*A*) MCF-7 cells were cultured at indicated pH conditions for 30 minutes, whole cell extract were prepared and analyzed for pAKT. (*B*) Phosphorylation of AKT under acidic environment is transient in MCF-7 cells but persistence in MDA-MB-231 cells. Cells were cultured at pH 6.6 for indicated times, whole cell extract were prepared and analyzed using indicated antibodies. (*C*) Acidic environment induces phosphorylation of PDK1 in MCF-7 cells. Cells were cultured at pH 6.6 for the indicated times, whole cell extract were prepared and analyzed by Western blotting using indicated antibodies.

Because NF-κB activation has been linked with activation of AKT, we investigated the effects of low pHe on the activity of AKT. *First*, exposure of cells to different pH conditions indicated that acidosis enhanced the phosphorylation of AKT at Ser^473^ (Fig. 3*A*). However, the total level of AKT was unaffected under the same conditions. *Second*, a time dependent increase in the phosphorylation of AKT at Ser^473^ (Fig. 3*B*) and Thr^308^ (Fig. S2*A*) was observed at low pH. Furthermore, normal pHe was unable to induce any change in the phosphorylation of AKT at Ser^473^ in MCF-7 cells (Fig. S2*B*). While the increase in AKT phosphorylation in MCF-7 cells was transient, MDA-MB-231 cells exhibited a persistent increase in AKT phosphorylation. We also investigated the phosphorylation of PDK1, a kinase upstream to AKT, and found that acidosis can activate PDK1 in a time-dependent manner in MCF-7 cells (Fig. 3*C*).

### Acidosis-induced activation of AKT and NF-κB is mediated through ROS generation

**Figure 4:**
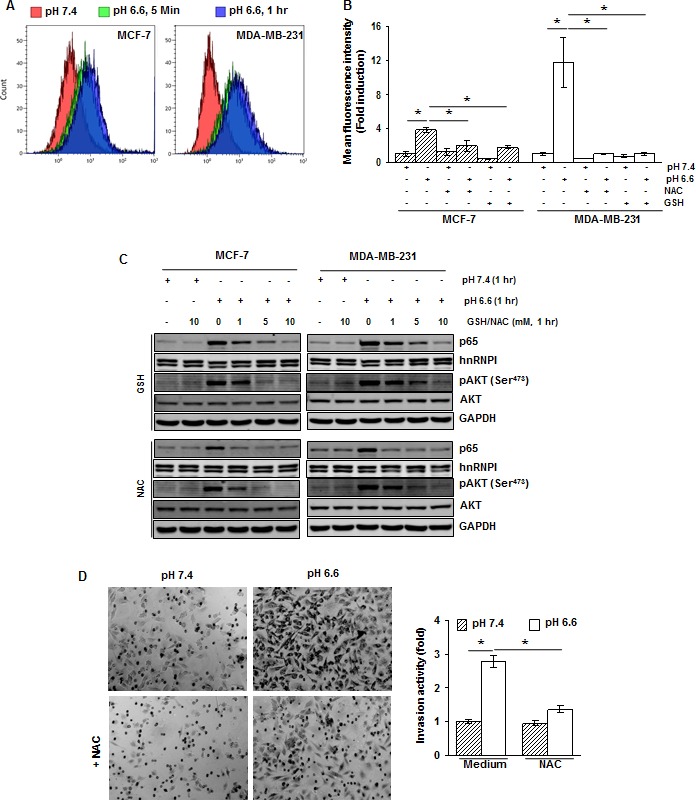
The acidosis-induced activation of NF-κB and AKT is mediated through ROS generation (*A*) Cells were cultured at pH 7.4 and pH 6.6 for 5 minutes and 1 hour. The intracellular ROS levels were measured using DCFH-DA by flow cytometry. (*B*) ROS generated by acidosis is suppressed by NAC and GSH. Cells were treated with NAC (10 mM) or GSH (10 mM) for 1 h, washed off, and then cultured at pH 7.4 or pH 6.6 for 1 hour. The intracellular ROS levels were measured using DCFH-DA by flow cytometry. (*C*) Cells were treated with indicated concentrations of GSH or NAC for 1 hour, washed off, and then cultured at pH 7.4 and pH 6.6 for 1 hour. The nuclear extracts and whole-cell extracts were analyzed by Western blotting for p65 and pAKT, respectively. (*D*) NAC suppresses acidosis induced invasion activity of MDA-MB-231 cells. Cells were pretreated with NAC (10 mM) for 1 hour, washed off, and then cultured at pH 7.4 or pH 6.6 for 48 hours. Invasion activity was measured using Matrigel invasion chambers.*, *p*< 0.05.

Studies during the past decades have suggested that there are numerous cross-talks among ROS generation, and NF-κB and AKT activation [17]. We therefore investigated if ROS is required for acidosis-induced AKT and NF-κB activation. *First*, acidosis was found to induce ROS production in both MCF-7 and MDA-MB-231 cells as early as 5 minutes of exposure (Fig. 4*A*). *Second*, the ROS scavengers, GSH and NAC, abrogated the acidosis-induced ROS production (Fig. 4*B*). For example, the generation of ROS was reduced from an 11.8-fold to a 1-fold in MDA-MB-231 cells (Fig. 4*B*).

To examine if acidosis-induced NF-κB and AKT activation is mediated through ROS generation, we pretreated the cells with 1, 5 and 10 mM GSH or NAC before culturing them under acidic conditions. We found that both GSH and NAC suppressed acidosis-induced NF-κB and AKT activation in a dose-dependent manner (Fig. 4*C*). The total level of AKT was unaffected under the same conditions.

To examine the possibility of involvement of ROS in acidosis-induced invasion activity, MDA-MB-231 cells were pretreated with 10 mM NAC, cultured under acidic conditions for 48 hours and then invasion assay was performed. The invasion activity was increased by a 2.8-fold under acidic conditions (Fig. 4*D*). However, when cells were pretreated with NAC, the activity was increased by only about a 1.4-fold. These results suggest that ROS plays a critical role in acidosis-induced invasion activity of breast cancer cells.

### Acidosis does not activate AKT and NF-κB in non-malignant breast cells

**Figure 5:**
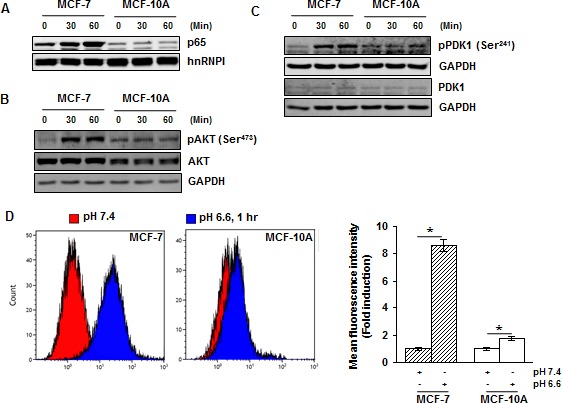
Acidosis does not activate NF-κB, AKT and PDK1, and induces minimal level of ROS in non-malignant cells (*A*) MCF-7 and MCF-10A cells were cultured under acidic conditions for the indicated time and the nuclear extracts were used to analyze p65 by Western blotting. (*B, C*) The whole-cell extracts were analyzed using indicated antibodies by Western blotting. (*D*) Cells were cultured at pH 7.4 and pH 6.6 for 1 hour and the intracellular ROS level was measured using DCFH-DA by flow cytometry. *, *p*< 0.05.

Next, we examined the effects of acidosis on non-malignant breast cells. The non-malignant breast cells (MCF-10A) and breast cancer cells (MCF-7) were cultured under low pH conditions for 30 and 60 minutes and NF-κB activation was examined. While acidosis induced NF-κB activation in MCF-7 cells, no activation was observed in MCF-10A cells (Fig. 5*A*). Similarly, we detected acidosis-induced AKT and PDK1 activation in MCF-7 cells but not in MCF-10A cells (Fig. 5*B* and 5*C*). We then investigated whether this cancer cell specific acidosis-induced NF-κB and AKT activation is due to a difference in ROS production. Both MCF-7 and MCF-10A cells were cultured under normal or low pH conditions for 60 minutes before measurement of ROS. While acidosis induced an 8.6-fold increase in ROS generation in MCF-7 cells, only a 1.8-fold increase was observed in MCF-10A cells (Fig. 5*D*). These results suggest that the effects of acidosis on NF-κB signaling pathway is cancer cell specific and ROS may contribute to this specificity.

### Acidosis induces AKT activation through thiol modification of PTEN

**Figure 6:**
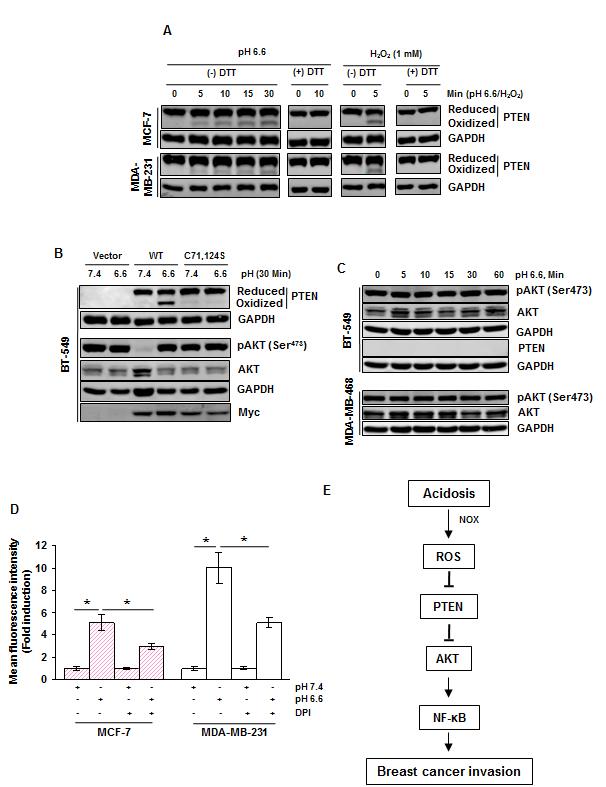
Acidosis oxidizes PTEN and presence of PTEN is required for acidosis induced AKT activation in breast cancer cells (*A*) MCF-7 and MDA-MB-231 cells were cultured under acidic conditions or treated with 1 mM H_2_O_2_ for the indicated time. The whole cell extract was then alkylated with NEM and subjected to nonreducing SDS-PAGE followed by Western blot analysis using indicated antibodies. For PTEN blot, upper band indicates the reduced form while lower band indicates the oxidized form of PTEN. (*B*) BT-549 cells transiently expressing wild-type and mutant (C^71^S, C^124^S) PTEN plasmids were exposed to acidosis for 30 minutes, whole cell extracts were prepared and analyzed for indicated proteins by Western blot analysis. (*C*) PTEN deficient cells (BT-549, MDA-MB-468) were exposed to acidosis for the indicated times, whole cell extracts were prepared and analyzed for indicated proteins by Western blot analysis. (*D*) The generation of ROS under acidic conditions is mediated through NOX in breast cancer cells. Cells were cultured at pH 7.4 or pH 6.6 without and with 20 μM DPI and analyzed for intracellular ROS levels using DCFH-DA by flow cytometry. (*E*) A schematic diagram showing the signaling pathways by which acidosis promotes breast cancer invasion.

The tumor suppressor PTEN, a negative regulator of AKT signaling, is known to undergo inactivation (oxidation) by ROS [18]. Since acidosis-induced ROS production and AKT phosphorylation were abrogated by ROS scavengers, we examined if activation of AKT is mediated through inactivation of PTEN. We found that acidosis induced PTEN oxidation in both MCF-7 and MDA-MB-231 cells (Fig. 6*A*). Furthermore, the reducing agent dithiothreitol (DTT) reversed the acidosis-induced PTEN oxidation in both cell lines, suggesting the possibility of involvement of cysteine residues. Consistent with previous reports, we also found that hydrogen peroxide oxidized PTEN which can be reversed by DTT. PTEN has two essential catalytic cysteine residues (Cys^71^ and Cys^124^) that are subject to thiol modification by ROS [18]. To determine if these residues are involved in acidosis-mediated PTEN oxidation and AKT phosphorylation, we transfected the PTEN deficient BT-549 cells with wild type Myc-PTEN or mutant Myc-PTEN with Cys^71^Ser and Cys^124^Ser. We detected acidosis-induced oxidation of PTEN in cells expressing wild type PTEN but not in those expressing mutant PTEN (Fig. 6*B*). Importantly, PTEN deficient cells expressed a high level of basal phospho-AKT (Fig. 6*B* and Fig S3) that was suppressed by the introduction of wild type PTEN but not by mutant PTEN (Fig. 6*B*). Furthermore, acidosis induced phosphorylation of AKT in cells expressing wild type PTEN but not in those expressing mutant PTEN. These results suggest that Cys^71^ and Cys^124^ residues of PTEN are essential for the acidosis-induced AKT phosphorylation. We also found that acidosis was unable to induce AKT phosphorylation in PTEN deficient BT549 and MDA-MB-468 cells (Fig. 6*C*), further supporting the role of PTEN in acidosis-mediated AKT phosphorylation.

### NADPH oxidase (NOX) contributes to acidosis-induced ROS generation

Finally, we determined whether the family of NADPH oxidase (NOX) is involved in acidosis-induced ROS because they are the most likely source of ROS produced in the cell [19]. We treated cells with diphenyleneiodonium (DPI), a pharmacological inhibitor of NOX, before culture under low pH conditions, and ROS was detected by the DCFH-DA method. We found that acidosis-induced ROS production was reduced from a 5.1-fold to a 2.9-fold in MCF-7 cells and from a 10-fold to a 5.1-fold by DPI in MDA-MB-231 cells (Fig. 6*D*), suggesting a role of NOX in acidosis-induced ROS generation.

## DISCUSSION

The extracellular pH (pHe) of solid tumors, such as breast tumors, is more acidic than that of normal tissue primarily due to high glycolysis and poor perfusion within tumors; pHe values could reach 6.2~6.8 [20]. In some extreme cases, interstitial pH values could even be as low as 5.8 [21]. Thus, these tumor cells have to employ various mechanisms to remove intracellular acids in order to maintain physiological pHi, including Na-driven proton extrusion, V-type ATPases, *Na*^+^/H^+^*exchangers* (NHEs) and those facilitated by carbonic anhydrases [22, 47]. As a result, pHe becomes more acidic, which is often harmful to normal cells. However, such low pHe may benefit tumor cells for their migration and invasion [23]. These findings suggest that tumor cells have adapted well to extracellular acidosis which, furthermore, may be used by tumor cells as a means to promote their invasion and metastasis [4].

Acidic tumor microenvironment has been shown to produce multiple effects on tumor cells and contribute to invasiveness and metastasis. For instance, acidosis-induced cell invasion has been reported in variety of cancer including melanoma [24, 25], cervical cancer [26], and prostate cancer [27]. Moreover, acidosis can activate NF-κB in melanoma [10], osteosarcoma [28], ovarian [11] as well as pancreatic, colon and prostate cancer [12], suggesting the importance of NF-κB in cell invasion. In support of this view, we show that acidosis induces NF-κB activation in breast cancer cells, as determined by Western blot, reporter gene assay and immunofluorescence staining. Moreover, this is a specific effect of acidosis-induced NF-κB because acidosis does not affect STAT3 activity. Finally, invasion activity is suppressed by gene silencing of NF-κB p65. As a result, acidosis induces the NF-κB target gene CXCR4. Notably, acidosis induced NF-κB p65 activation was found to be more in MCF-7 cells in comparison to MDAMB-231 cells. However, acidosis does not promote invasiveness in MCF-7 cells (unpublished observations). Although NF-κB p65 is one of the important contributing molecules facilitating invasiveness of breast cancer cells, there are several other factors that can mask the effects of p65. For example, MCF-7 cells lack CD44^+^/CD24^−^ subpopulations, a marker associated with invasiveness, whereas MDA-MB-231 cells have high CD44^+^/CD24^−^ subpopulations [29]. The expression levels of other pro-invasive genes such as interleukin (IL)-8, and urokinase plasminogen activator (uPA) are higher in MDA-MB-231 cells in comparison to MCF-7 cells [30]. Furthermore, MCF-7 cells express high level of E-cadherin, an epithelial adhesion molecule that prevents invasiveness of carcinoma cells, whereas MDA-MB-231 cells lack E-cadherin. It is likely that the effects of these molecules overweigh the effects of NF-κB p65 and thus MCF-7 cells remains non-invasive.

However, the underlying mechanism of how acidosis induces intracellular signaling pathways, particularly NF-κB activation, is poorly understood. The present study demonstrates a series of signaling events upon acidosis (Fig 6*E*). At the top of acidosis-induced signaling is ROS production, which inactivates PTEN through oxidation. As a major phosphatase and inhibitor, PTEN counteracts the action of PI3K. This may explain why PDK1 and AKT are activated upon acidosis. Activated AKT serves as an upstream signaling molecule for NF-κB through IκBα kinase (IKK) and IκBα [31]. Degradation of IκBα causes NF-κB to move into the nucleus to regulate a specific set of genes.

In this regard, we showed that acidosis-induced IκBα degradation is concomitant with IκB phosphorylation, p65 phosphorylation and p65 nuclear translocation. Although numerous kinases are involved in NF-κB activation, IKK complex is an important site for the integration of signals that regulate the NF-κB pathway [13, 32] and AKT reportedly functions as an immediate upstream molecule of IKK [33]. At upstream of AKT, PDK1 can serves as a kinase for AKT and it is activated by phosphatidylinositol-4,5-trisphosphate (PI(3,4,5)P3), a second messenger that is controlled by phosphatidylinositol 3-kinase (PI3K) [34]. This allows the recruitment of PDK1 and AKT into the plasma membrane [35] and subsequent activation of AKT by PDK1.

On the other hand, PTEN, as a phosphatase, is able to remove phosphate attached to the 3′-hydroxyl group of the PI(3,4,5)P3 [36]. Therefore, inhibition of PTEN elevates intracellular level of PI(3,4,5)P3, leading to AKT activation. Cells at pH 6.6 in 5 minutes generate an oxidized (inactive) form of PTEN that migrates faster on the non-reducing gel. The crystal structure of human PTEN has revealed the presence of two essential adjacent cysteine residues at 71 and 124 positions in its catalytic domain that are prone to oxidation (inactivation) by ROS to form a disulfide bond [18]. Dithiothreitol and mutations of Cys^71^ and Cys^124^ of PTEN to Ser block acidosis-induced PTEN oxidation. The introduction of wild type PTEN in PTEN deficient cells significantly reduces the basal level of pAKT, whereas mutant PTEN has a minimal effect. In agreement with these observations, catalytically active form of PTEN has been shown to suppress basal activation of AKT [37, 38]. Furthermore, acidosis enhances the level of pAKT in cells expressing wild type PTEN but not in those expressing mutant PTEN. We speculate that acidosis-mediated ROS production induces disulfide bond formation between Cys^71^ and Cys^124^ of PTEN that causes the loss of its phosphatase activity, resulting in enhanced AKT phosphorylation. Finally, acidosis is unable to induce AKT activation in PTEN deficient cells, further indicating that PTEN is required for acidosis-induced AKT activation.

Our study suggests that ROS serves as a top signal in response to acidosis. *First*, acidosis induces ROS production in both MCF-7 and MDA-MB-231 cells. *Second*, ROS scavengers are able to suppress ROS production. *Third*, AKT and NF-κB activation are also suppressed by ROS scavengers. *Fourth*, ROS scavengers suppress acidosis-induced invasion activity. However, detail mechanisms by which acidosis induces ROS remain to be determined yet. Since the NADPH oxidase (NOX) inhibitor DPI can suppress acidosis-induced ROS production, it is possible that breast cancer cells generate ROS under acidic conditions in part through NOX. Our observation is not consistent with a previous study that acidosis-induced ROS generation is independent of NOX in prostate cancer cells [39]. It remains to be determined whether this discrepancy is due to the use of different types of cancer cells. Since NOX is present in cell's plasma membrane [40], plasma membrane appears to be major site of ROS production under acidic conditions. Thus, it would be interesting to determine whether NOX interacts with molecular sensors such as proton-sensing G-protein coupled receptors (GPCRs) or acid-sensing ion channels (ASICs) [22] to induce ROS production.

However, this acidosis-mediated signaling pathway we demonstrate here does not exist in all tumors. For instance, PTEN mutations occurs in a variety of cancer types including breast cancer [41]. Thus, a question is whether PTEN mutant tumor cells are still responsive to acidosis. If so, what are alternative pathways? We found that acidosis also activates ERK1/2, which can be suppressed by both GSH and NAC in MCF-7 cells (Gupta et el., unpublished). Hence, it would be interesting to determine whether activation of ERK1/2 can bypass AKT to activate NF-κB or a further downstream factor. Given the heterogeneity of tumor cells, it is likely that different types of tumor cells may have different intracellular signaling pathways in response to acidosis. Therefore, further characterization of acidosis-induced signaling in various types of tumor cells will provide more comprehensive insight into acidosis-induced cell invasion.

One of the most interesting findings is cancer specific acidosis-induced ROS production and activation of AKT and NF-κB. ROS is significantly high in breast cancer MCF-7 cells compared to non-malignant MCF-10A cells. Due to the unique metabolic profile, tumor cells may have elevated activity of NOX that may lead to enhanced ROS generation in these cells in comparison with normal cells. It is possible that a cellular threshold of ROS is critical to activation of above key events [42]. Because of their inherent capability to survive under oxidative stress, cancer cells can accumulate ROS to achieve a high cellular threshold under acidic conditions. On the other hand, normal or non-malignant cells may lack such a mechanism so that they cannot survive well under acidosis. Therefore, this high level of ROS in cancer cells may help them to achieve the acidosis-induced signaling cascades [43]. Evidently, this selectivity may provide a great opportunity for intervention. Of course, studies are underway to widen the relevance of these observations using multiple tumor types and non-malignant cells.

## MATERIALS AND METHODS

### Reagents

DMEM/F12 and RPMI 1640 media were obtained from Lonza (Walkersville, MD). The fetal bovine serum (FBS), aprotonin, leupeptin, benzamidine hydrochloride, diphenyleneiodonium chloride (DPI), 2′,7′-dichlorofluorescin diacetate (DCFH-DA), N-acetyl-L-cysteine (NAC), L-glutathione reduced (GSH), TNF-α, N-ethylmaleimide (NEM), dithiothreitol, and β-glycerophosphate disodium salt hydrate were obtained from Sigma-Aldrich (St. Louis, MO). The antibodies were obtained from following sources: NF-κB-p65, poly (ADP-ribose) polymerase (PARP), PTEN, PDK1, STAT3, pp65 (Ser^536^), pIκBα (Ser^32/36^), pAKT (Ser^473^ and Thr^308^), pPDK1 (Ser^241^), and pSTAT3 (Ser^727^) from Cell Signaling (Danvers, MA); AKT from Santa Cruz Biotechnology (Santa Cruz, CA); IκBα from Imgenex (San Diego, CA); CXCR4 from Abcam (Cambridge, MA); GAPDH from ProteinTech (Chicago, IL). Antibody against hnRNP I was custom made. The goat anti-rabbit Alexafluor 488 was obtained from invitrogen (Carlsbad, CA) while secondary antibodies conjugated with IRDye 800CW or IRDye 680 were purchased from LI-COR Biosciences (Lincoln, NE). The sources of other materials were: BD Matrigel invasion chamber from BD Biosciences (San Jose, CA); MG-132 from Calbiochem (Billerica, MA); phusion enzyme for PCR reactions and cloning purpose, and H_2_O_2_ from Fischer Scientific (Pittsburgh, PA); RNAfectin from Applied Biological Materials (Vancouver, Canada); and luciferase assay kit from Promega (Madison, WI). PCR primers were purchased from IDT (Coralville, IA) (Supplementary Table S1). siRNA sources were: control siRNA from Fisher Scientific (Pittsburgh, PA); p65 siRNA from Cell Signaling (Danvers, MA).

### Cell lines

All cell lines were obtained from American Type Culture Collection (Manassas, VA) otherwise stated. MCF-7, MDA-MB-231, and BT-549 cells were cultured in RPMI-1640 medium with 10% FBS and 2 mM glutamine. MDA-MB-468 cells were cultured in DMEM/F12 with 10% FBS and 2 mM glutamine while SUM-149 cells were cultured in Ham's F12 containing 10% FBS, 5 μg/mL insulin and 1μg/mL hydrocortisone. MCF-10A cells were cultured in DMEM/F-12 with 5% FBS, 1% L-glutamine, 10 ng/mL EGF, 0.5 μg/mL hydrocortisone, and 10 μg/mL insulin. All culture media were supplemented with 100 units/mL penicillin and 100 μg/mL streptomycin.

### Immunofluorescence staining and confocal microscopy

To determine the subcellular localization of NF-κB p65 in cells grown under normal and low pH medium, an immunofluorescence staining was performed as described previously [44]. In brief, cells were fixed with 4% paraformaldehyde (pH 7.2), permeabilized with Triton X-100, and blocked with 2.5% bovine serum albumin. Cells were then incubated with rabbit polyclonal anti-p65 (1:100) at 4°C overnight, followed with goat anti-rabbit immunoglobulin G Alexa Fluor 488 (1:100) for 1 h. After nuclear staining with propidium iodide (1 μg/mL) and mounting, the cells were examined under Nikon C1 confocal scanning microscope at Imaging Core of Center for Psychiatric Neuroscience at UMMC.

### Invasion assay

Cell invasion activity was determined *in vitro* using BD BioCoat tumor invasion system from BD Biosciences (San Jose, CA), which contains an 8-μm polyethylene terephthalate membrane with a thin layer of reconstituted Matrigel basement membrane matrix. Cells (2 × 10^4^) in 500 μL medium were added to the wells and incubated at 37°C in an incubator equipped with 5% CO_2_. After 24 hours, cells remaining in the upper membrane surface were removed with a cotton swab, whereas the cells on the lower surface were fixed in methanol, stained with crystal violet and counted as described before [45].

### Plasmid construction

To construct NF-κB-luciferase reporter system, 5 copies of NF-κB response element in front of minimal promoter (TATA) was custom synthesized as a gBlock (IDT) which was then used as a template for PCR amplification using primers pGL3-B-Xho1-5.1 and TK mini-Luc-Nco1-3.1 (Supplementary Table S1). The PCR product was cloned into the luciferase reporter vector (PGL3 basic) at XhoI and NcoI sites. Wild type PTEN and mutant PTEN (C71S and C124S) were cloned into pCDH-CMV-copGFP (System Biosciences, Mountain View, CA). For wild type PTEN, we used primers PTEN-Myc-R1-5.1 and PTEN-Not1-3.1, tagged with N-terminal Myc. For mutant PTEN, we used primers PTEN-C71S-5.1, PTEN-C71S-3.1, PTEN-C124S-5.1 and PTEN-C124S-3.1 to introduce two mutant sites (C71S and C124S). The cloned mutant PTEN was also tagged with N-terminal Myc. All PCR products were verified by DNA sequencing.

### NF-κB-dependent reporter assay

To examine the effect of acidosis on NF-κB-dependent reporter gene transcription, MCF-7 cells were transiently transfected with the NF-κB reporter plasmid before culture under normal or low pH medium. The luciferase activity was measured using whole cell lysate and a luciferase assay kit following the manufacturer's instructions [46]. The firefly luciferase activity was normalized to renilla luciferase activity.

### Transfection

Cells were transfected with siRNAs using RNAfectin reagent essentially following the manufacturer's instructions as described before [45].

### Measurement of intracellular ROS

We used membrane-permeable DCFH-DA dye to measure intracellular ROS generation by flow cytometry. Cells were cultured under normal or low pH medium in the presence of dye and the assay was conducted according to a previously published protocol [48].

### Western blot

The protein extract from cytoplasmic, nuclear and whole cell extracts were prepared, and Western blot analysis was carried out as described previously [49].

### Preparation of nuclear, cytoplasmic, and whole cell extracts

The nuclear, cytoplasmic and whole cell extracts were prepared as described previously [50] and the protein concentration in these fractions was determined by the Bradford method.

### Identification of reduced and oxidized forms of PTEN

To detect oxidized and reduced forms of PTEN, we followed a method described previously [18]. In brief, whole cell protein extract was prepared from normal and treated cells in a whole cell lysis buffer containing 40 mM NEM. An equal amount of protein was resolved by 10% SDS-PAGE under non-reducing conditions; oxidized and reduced forms of PTEN were detected by Western blotting.

### Statistical methods

Different parameters were monitored in normal and treated groups. The two groups were compared by an unpaired Student's t test. A value of *P*< 0.05 was considered statistically significant.

## SUPPLEMENTARY MATERIAL FIGURES




