# Supplementary material for: Acidosis promotes invasiveness of breast cancer cells through ROS-AKT-NF-κB pathway
Source: Oncotarget. 2014 Sep 25;5(23):12070–82. doi: 10.18632/oncotarget.2514 (PMC4322981; doi:10.18632/oncotarget.2514)
Supplement: Supplementary file 1 [file oncotarget-05-12070-s001.pdf]

Acidosis promotes invasiveness of breast cancer cells through ROS-AKT-NF-κB pathway

Supplementary Material

Table S1 Oligonucleotides used for gene cloning in this study

5x NFkB-TK mini
CGTGCTAGCCCGGGCTCGAGGGGAATTTCCGGGGACTTTCCGGGAATTTCCGGGGACTTTCCGGGAATTT
CCGAACACGCAGATGCAGTCGGGGCGGCGCGGTCCGAGGTCCACTTCGCATATTAAGGTGACGCGTGTGG
CCTCGAACACCGA

pGL3-B-Xho1-5.1	CGTGCTAGCCCGGGCTCGAG
TKmini-Luc-Nco1-3.1	TTGGCGTCTTCCATGGTGGCTCGGTGTTCGAGGCCACACG
PTEN-Myc-R1-5.1	CCATGGAGGCCCGAATTCTGACAGCCATCATCAAAGAG
PTEN-Not1-3.1	TCGCAGATCCTTGCGGCCGCTCAGACTTTTGTAATTTGTG
PTEN-C71S-5.1	TTACAAGATATACAATCTTAGTGCTGAAAGACATTATGAC
PTEN-C71S-3.1	GTCATAATGTCTTTCAGCACTAAGATTGTATATCTTGTAA
PTEN-C124S-5.1	ATCATGTTGCAGCAATTCACAGTAAAGCTGGAAAGGGACG
PTEN-C124S-3.1	CGTCCCTTTCCAGCTTTACTGTGAATTGCTGCAACATGAT

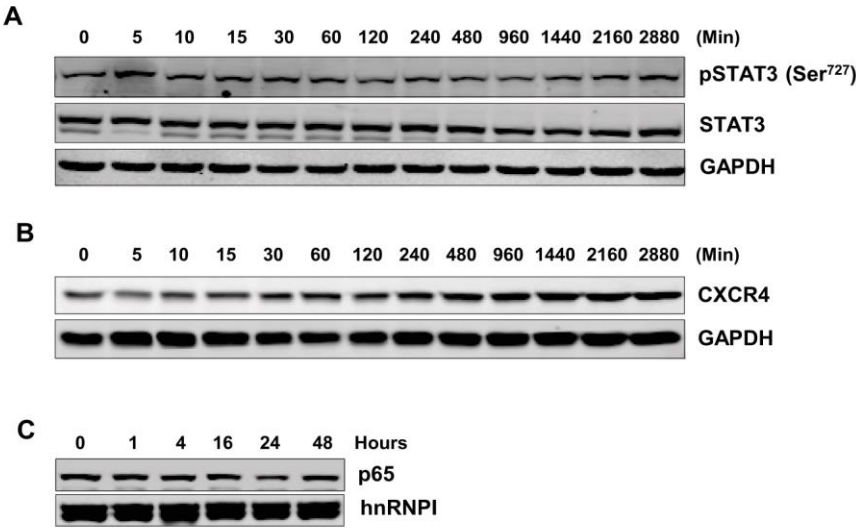

Figure S1: Acidosis does not activate STAT3 but induces CXCR4 in breast cancer cells. (A, B) MCF-7 cells were cultured at pH 6.6 for the indicated times, whole cell extracts were prepared and analyzed for pSTAT3, STAT3 and CXCR4 by Western blotting. GAPDH was used as an internal control. (C) MCF-7 cells were cultured at pH 7.4 for the indicated times, nuclear extracts were prepared and analyzed for NF-KB-p65 by Western blotting.

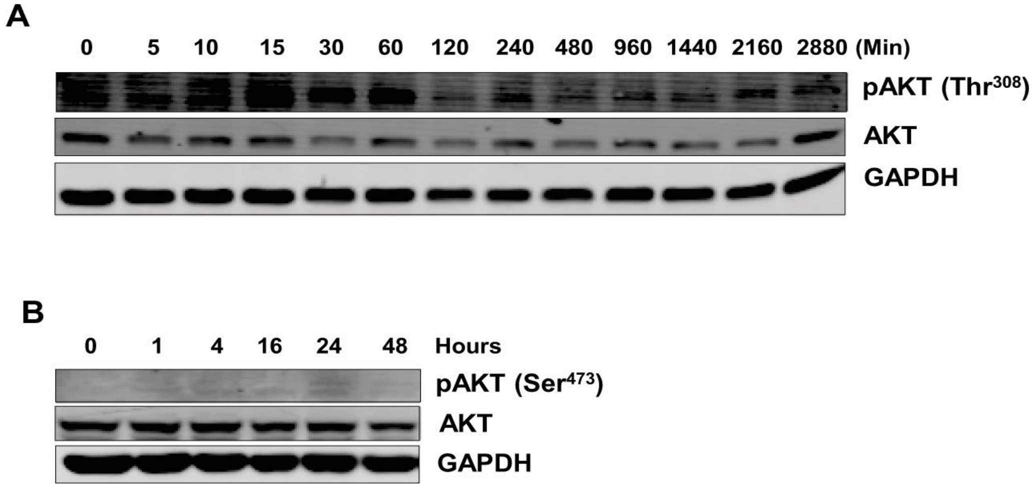

Figure S2: Acidosis induces phosphorylation of AKT at Thr^{308}. (A) MCF-7 cells were cultured at pH 6.6 for indicated times, whole cell extract were prepared and analyzed using indicated antibodies by Western blotting. (B) MCF-7 cells were cultured at pH 7.4 for indicated times, whole cell extract were prepared and analyzed using indicated antibodies by Western blotting.

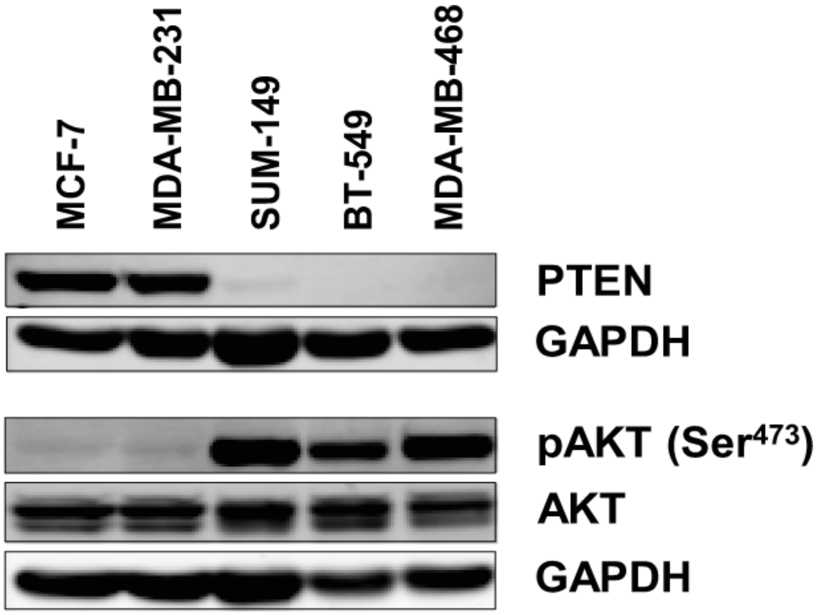

Figure S3: Constitutive activation of AKT in PTEN deficient cells. The whole cell extracts were prepared from indicated cell lines and analyzed for indicated proteins by Western blot analysis.
