# Supplementary material for: Acidosis promotes invasiveness of breast cancer cells through ROS-AKT-NF-κB pathway
Source: Oncotarget. 2014 Sep 25;5(23):12070–82. doi: 10.18632/oncotarget.2514 (PMC4322981; doi:10.18632/oncotarget.2514)
Supplement: Supplementary file 2 [file oncotarget-05-12070-s002.docx]

Table Si Oligonuczieotides used for gene czioning in this study

5x NFkB-TK mini

CGTGCTAGCCCGGGCTCGAGGGGAATTTCCGGGGACTTTCCGGGAATTTCCGGGGACTTTCCGGGAATTT

CCGAACACGCAGATGCAGTCGGGGCGGCGCGGTCCGAGGTCCACTTCGCATATTAAGGTGACGCGTGTGG

CCTCGAACACCGA

pGL3-B-Xhoi-5. i CGTGCTAGCCCGGGCTCGAG

TKmini-Luc-Nooi-3 . i TTGGCGTCTTCCATGGTGGCTCGGTGTTCGAGGCCACACG

PTEN-Myc-Ri-5. 1 CCATGGAGGCCCGAATTCTGACAGCCATCATCAAAGAG

PTEN—Noti-3. 1 TCGCAGATCCTTGCGGCCGCTCAGACTTTTGTAATTTGTG

PTEN-C7i5-5 . 1 TTACAAGATATACAATCTTAGTGCTGAAAGACATTATGAC

PTEN-C71S-3. 1 GTCATMTGTCTTTCAGCACTAAGATTGTATATCTTGTAA

PTEN-C124S-5. 1 ATCATGTTGCAGCAATTCACAGTAAAGCTGGAAAGGGAOG

PTEN—Ci24S-3. 1 CGTCCCTTTCCAGCTTTACTGTGAATTGCTGCAACATGAT
